# Maternal and fetal/neonatal outcomes of pregnancies complicated by pulmonary hypertension: a retrospective study of 154 patients

**DOI:** 10.1016/j.clinsp.2023.100194

**Published:** 2023-04-27

**Authors:** Chengtian Lv, Lichan Wu, Guangyuan Liao, Yuwen Huang, Jingyi Chen, Shuyi Jiang, Dunjin Chen, Yuanmei Gao

**Affiliations:** a Department of Respiratory and Critical Care Medicine, Guangdong Provincial Key Laboratory of Major Obstetric Diseases, The Third Affiliated Hospital of Guangzhou Medical University, Guangzhou, China; b Department of Critical Care Medicine, Guangdong Provincial Key Laboratory of Major Obstetric Diseases, The Third Affiliated Hospital of Guangzhou Medical University, Guangzhou, China; c The Third Clinical College of Guangzhou Medical University, Guangzhou, China; d Department of Obstetrics and Gynecology, Department of Fetal Medicine and Prenatal Diagnosis, Guangdong Provincial Key Laboratory of Major Obstetric Diseases, The Third Affiliated Hospital of Guangzhou Medical University, Guangzhou, China

**Keywords:** Pregnancy, Pulmonary hypertension, Pregnancy outcomes, Maternal death

## Abstract

**Objectives:**

To determine the main clinical and demographic outcomes related to Pulmonary Hypertension (PH) and adverse obstetric and fetal/neonatal outcomes.

**Methods:**

This study retrospectively analyzed the medical record data of 154 patients with PH who were admitted to the Third Affiliated Hospital of Guangzhou Medical University between January 2011 and December 2020.

**Results:**

According to the severity of elevated Pulmonary Artery Systolic Pressure (PASP), 82 women (53.2%) were included in the mild PH group, 34 (22.1%) were included in the moderate PH group, and 38 (24.7%) were included in the severe PH group. There were significant differences in the incidence of heart failure, premature delivery, Very-Low-Birth-Weight (VLBW) infants, and Small-for-Gestational-Age (SGA) infants among the three PH groups (p < 0.05). Five (3.2%) women died within 7-days after delivery, 7 (4.5%) fetuses died in utero, and 3 (1.9%) neonates died. The authors found that PASP was an independent risk factor for maternal mortality. After adjustment for age, gestational weeks, systolic blood pressure, Body Mass Index (BMI), mode of delivery, and anesthesia, the risk of maternal mortality in the severe PH group was 20.21 times higher than that in the mild-moderate PH group (OR = 21.21 [95% CI 1.7∼264.17]), p < 0.05. All 131 (85.1%) patients were followed up for 12 months postpartum.

**Conclusions:**

The authors found that the risk of maternal mortality in the severe PH group was significantly higher than that in the mild-moderate group, highlighting the importance of pulmonary artery pressure screening before pregnancy, early advice on contraception, and multidisciplinary care.

## Highlights

• Pregnant women with severe pulmonary hypertension have a higher risk of mortality.
• Pulmonary artery systolic pressure is associated with maternal mortality.
• Screening before pregnancy and multidisciplinary monitoring are crucial.

## Introduction

Pulmonary Hypertension (PH) during pregnancy is a pathophysiological condition caused by pulmonary vascular remodeling and a continuous increase in pulmonary vascular resistance. Severe PH can lead to right heart failure and even death.^1^ The prevalence of PH in pregnant women is 97 cases per million, and the mortality rate is as high as 25%–26%. The mortality rate of fetuses or newborns is 7%–13%.^2^, ^3^, ^4^, ^5^, ^6^, ^7^ The main cause of maternal death in pregnancy with PH is that the right ventricle and pulmonary vascular system are unable to adapt to changes in the cardiovascular system during pregnancy and the postpartum period. Furthermore, PH seriously affects the development of embryos and fetuses, thus increasing the risk of abortion, premature delivery, and intrauterine fetal growth restriction.^6^ This condition seriously threatens the lives and safety of pregnant women and their fetuses. According to expert guidelines, pregnancy is not recommended for women with PH, so these women should terminate the pregnancy in the early stage.^8^^,^^9^ In recent years, the increasing number of women with high-risk pregnancies has led to serious challenges in clinical work, and research on pregnancies complicated with PH has attracted increasing attention. Pregnancy outcomes are different in women with different clinical classifications of PH and differ among PH patients with different parities. However, studies on the pregnancy outcomes of pregnant women with different severities of PH are rare. This study aimed to determine the relationship between the severity of PH and adverse obstetric and fetal/neonatal outcomes; a retrospective analysis of 154 patients who were treated at the Third Affiliated Hospital of Guangzhou Medical University in the last 10 years was performed. This study will provide a reference for the clinical diagnosis and treatment of these kinds of high-risk pregnancy patients.

## Materials and methods

### Study population and design

**Fig. 1:**
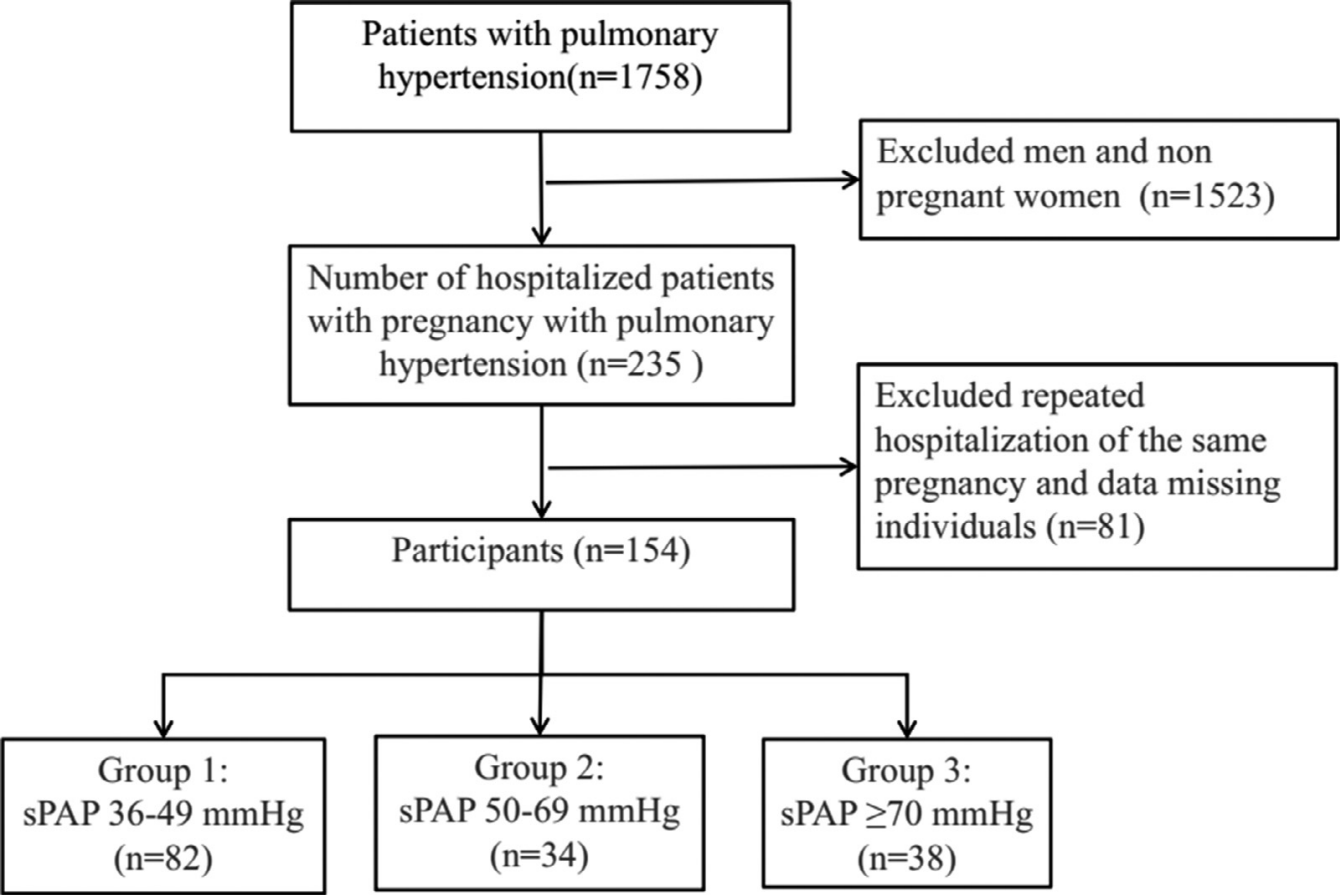
Participant inclusion flowchart.

The study was conducted according to the guidelines of the Declaration of Helsinki and approved by the institutional review board of the Third Affiliated Hospital of Guangzhou Medical University, with ethics approval ID [2022] NO. 030. (Protocol code 30 and date of approval 24 April 2022). The electronic medical record system included the records of 1758 patients with PH who were treated in the hospital from January 2011 to December 2020. After the screening, 154 patients diagnosed with PH during pregnancy were ultimately selected as the research participants (see Fig. 1 for details). Descriptive analyses of the effect of PH on maternal and fetal/neonatal outcomes were performed.

### Diagnostic, inclusion and exclusion criteria

PH refers to a mean Pulmonary Artery Pressure (mPAP) ≥ 25 mmHg (1 mmHg = 0.133 kPa) measured by Right Heart Catheterization (RHC) at sea level and at rest.^7^ Since the study participants were pregnant women, the tricuspid regurgitation pressure gradient determined by echocardiography was used to indirectly evaluate Pulmonary Artery Systolic Pressure (PASP), as is often performed in obstetrics clinics.^10^

In this study, the inclusion criteria were as follows: all subjects had undergone cardiac echocardiography, met the diagnostic criteria of echocardiography, had PH values determined according to the clinical diagnostic criteria, and had a PASP confirmed to be more than 35 mmHg by echocardiography.^11^, ^12^, ^13^ The exclusion criterion was patients with elevated PASP caused by outflow tract obstruction/pulmonary stenosis.

### Grouping method

The patients were classified according to the severity of elevated PASP: those with PASP of 36–49 mmHg were in the mild PH group, those with PASP of 50–69 mmHg were in the moderate PH group, and those with PASP ≥70 mmHg were in the severe PH group.^6^^,^^11^ The pregnant women were also classified according to the etiology of PH, including idiopathic Pulmonary Arterial Hypertension (iPAH), Congenital Heart Disease-related Pulmonary Arterial Hypertension (CHD-PAH), Pulmonary Arterial Hypertension caused by other diseases (oPAH), PH caused by left ventricular systolic dysfunction, and Left Heart Disease-related Pulmonary Hypertension (LHD-PH).^14^

### Outcome measures

The maternal outcome is maternal death. The fetal/neonatal outcome is fetal death in utero or neonatal death (within 28 days after birth).

### Statistical analysis

All analyses were performed using SPSS statistical software (version 25.0) (SPSS Inc., Chicago, IL, USA). The authors compared and analyzed the baseline characteristics and maternal and fetal/neonatal outcomes of participants in the mild, moderate, and severe PH groups. Categorical data are presented as frequencies and percentages, and Chi-Square tests were used for comparison. If there were fewer than five cases in a group, Fisher's exact test was used. The normality of continuous data was checked with Kolmogorov-Smirnov tests and presented either as the mean±standard deviation or as the median and first and third Quartiles (Q1–Q3) as appropriate. Differences among groups were assessed using one-way ANOVA or, in the case of nonnormality, using the Kruskal-Wallis nonparametric test. Multivariate logistic regression analysis was applied to estimate the Odds Ratio (OR) and 95% Confidence Interval (CI) of maternal death for the severe PH group compared with the mild-moderate PH group. The OR in multivariate logistic regression analysis was adjusted for confounding factors. Differences were considered significant when the p-value was less than 0.05.

## Results

### Baseline characteristics

In this study, 154 patients (Table 1) (aged 21–46 years, mean age, 30.9±5.1 years) were newly diagnosed with PH during pregnancy and had a gestational period of 6–41 weeks (mean gestational period, 33.1±7.4 weeks). According to the severity of elevated PASP, 82 women (53.2%) were included in the mild PH group, 34 (22.1%) were included in the moderate PH group, and 38 (24.7%) were included in the severe PH group.

**Table 1:** Baseline characteristics and maternal outcomes in pregnancies complicated by pulmonary hypertension (PH).

Variables	Total (n = 154)	Mild PH group (n = 82)	Moderate PH group (n = 34)	Severe PH group (n = 38)	p-value*
	n (%), Mean ± SD or median (IQR)				
Baseline characteristics					
Age (years), Mean ± SD	30.9 ± 5.1	31.2 ± 5.1	32.5 ± 4.8	29.1 ± 5.1	0.015^c^
BMI (kg/m^2^), Mean ± SD	25.9 ± 4.7	26.7 ± 5.2	25.7 ± 3.9	24.4 ± 3.9	0.044^c^
Systolic BP (mmHg), Mean ± SD	124.7 ± 20.2	127.8 ± 21.5	126.0 ± 17.0	116.9 ± 18.1	0.019^c^
Delivery week, Median (IQR)	35.5 (31.0, 38.0)	37.0 (34.0, 39.0)	33.5 (28.5, 37.0)	32.0 (29.0, 36.8)	<0.001^d^
End-systolic RA area (cm^2^), Median (IQR)	21.2 (17.2, 24.2)	18.6 (15.9, 22.3)	22.0 (16.9, 25.6)	25.0 (21.3, 29.3)	<0.001^d^
End-systolic RA area > 18 cm^2^, n (%)	106 (68.8)	50 (61)	23 (67.6)	33 (86.8)	0.017^a^
Etiological classification of PH					<0.001^b^
iPAH, n (%)	6 (3.9)	1 (1.2)	0 (0)	5 (13.2)	
CHD-PAH, n (%)	41 (26.6)	16 (19.5)	7 (20.6)	18 (47.4)	
oPAH, n (%)	45 (29.2)	35 (42.7)	8 (23.5)	2 (5.3)	
LHD-PH, n (%)	62 (40.3)	30 (36.6)	19 (55.9)	13 (34.2)	
NYHA class, n (%)					<0.001^a^
I–II	112 (72.7)	70 (85.4)	23 (67.6)	19 (50)	
III–IV	42 (27.3)	12 (14.6)	11 (32.4)	19 (50)	
Place of residence, n (%)					0.019^a^
Urban	79 (51.3)	48 (58.5)	19 (55.9)	12 (31.6)	
Rural	75 (48.7)	34 (41.5)	15 (44.1)	26 (68.4)	
Maternal admission to ICU, n (%)	54 (35.1)	13 (15.9)	14 (41.2)	27 (71.1)	<0.001^a^
Maternal outcomes					
Delivery mode, n (%)					
Vaginal delivery	31 (20.1)	24 (29.3)	2 (5.9)	5 (13.2)	0.008^b^
Cesarean section	108 (70.1)	52 (63.4)	27 (79.4)	29 (76.3)	0.145^a^
Anesthesia mode					
General anesthesia, n (%)	52 (33.8)	19 (23.2)	13 (38.2)	20 (52.6)	0.005^a^
intraspinal anesthesia, n (%)	56 (36.4)	33 (40.2)	14 (41.2)	9 (23.7)	0.173^a^
Maternal death (Postpartum ≤7 days), n (%)	5 (3.2)	1 (1.2)	0 (0)	4 (10.5)	0.034^b^
12-months all-cause death	8 (5.2)	1 (1.2)	0 (0)	7 (18.4)	0.001^b^
Other complications					
Uterine atony, n (%)	26 (16.9)	14 (17.1)	7 (20.6)	5 (13.2)	0.701^a^
Postpartum hemorrhage, n (%)	16 (10.4)	10 (12.2)	4 (11.8)	2 (5.3)	0.52^b^
Heart failure, n (%)	23 (14.9)	7 (8.5)	5 (14.7)	11 (28.9)	0.014^a^
Multiple organ dysfunction, n (%)	8 (5.2)	3 (3.7)	1 (2.9)	4 (10.5)	0.294^b^
Pulmonary thromboembolism, n (%)	4 (2.6)	0 (0)	1 (2.9)	3 (7.9)	0.029^b^

BMI, Body Mass Index; BP, Blood Pressure; NYHA, New York Heart Association; PASP, Pulmonary Artery Systolic pressure; RA, Right Atrium; CHD-PAH, Congenital Heart Disease-Related Pulmonary Arterial Hypertension; iPAH, Idiopathic Pulmonary Arterial Hypertension; LHD-PH, Pulmonary Hypertension caused by Left Heart Disease; oPAH, Pulmonary Arterial Hypertension caused by Other Diseases.

* p-value of difference among the mild, moderate, and severe PH groups.

a Chi-square test.

b Fisher's Exact Test.

c One-way ANOVA.

d Kruskal-Wallis nonparametric test.

In this study, the average ages of patients in the mild, moderate, and severe PH groups were 31.2 ± 5.1, 32.5 ± 4.8, and 29.1 ± 5.1 years, respectively. There were significant differences among the three groups (p < 0.05). The median right atrial area (IQR) at the end of the systolic period in the mild, moderate, and severe PH groups was 18.6 (15.9, 22.3), 22.0 (16.9, 25.6), and 25.0 cm^2^ (21.3, 29.3), respectively, and there were significant differences among the three groups (p < 0.001). There were 50 patients (60%, 50/82), 23 patients (67.6%, 23/34), and 33 patients (86.8%, 33/38) with a right atrial area > 18 cm^2^ at the end of cardiac contraction in the mild, moderate, and severe PH groups, respectively, and the difference among the groups was statistically significant (p = 0.017). The mean Body Mass Index (BMI) of patients with mild, moderate, and severe PH was 26.7 ± 5.2, 25.7 ± 3.9, and 24.4 ± 3.9 kg/m^2^, respectively, and the difference among the groups was statistically significant (p = 0.044). The mean systolic Blood Pressure (systolic BP) in patients with mild, moderate, and severe PH was 127.8±21.5 mmHg, 126.0 ± 17.0 mmHg, and 116.9 ± 18.1 mmHg, respectively, and there were significant differences among the three groups (p < 0.05). The median delivery week (IQR) in the mild, moderate, and severe PH groups was 37.0 (34.0, 39.0), 33.5 (28.5, 37.0), and 32.0 weeks (29.0, 36.8), respectively, and there were significant differences among the three groups (p < 0.001).

In the mild PH group, 85.4% of the pregnant women had cardiac function grades of I–II, and 14.6% had cardiac function grades of III–IV; in the moderate PH group, 67.6% of the pregnant women had cardiac function grades of I–II, and 32.4% had grades of III–IV; and in the severe PH group, 50% of the patients had cardiac function grades of I–II and III–IV, respectively, and there were significant differences among the three groups (p < 0.05). The proportions of pregnant women in the mild, moderate, and severe PH groups admitted to the ICU were 15.9%, 41.2%, and 71.1%, respectively, and there were significant differences among the three groups (p < 0.05).

Among the 154 pregnant women in this study whose pregnancies were complicated by PH, according to the etiological classification, there were six cases of iPAH (3.9%), 41 cases of CHD-PAH (26.6%), 45 cases of oPAH (29.2%), and 62 cases of LHD-PH (40.3%). oPAH (42.7%) accounted for the largest proportion of pregnant women in the mild PH group, and LHD-PH (55.9%) accounted for the largest proportion in the moderate PH group. However, CHD-PAH (47.4%) accounted for the largest proportion of pregnant women in the severe PH group, mainly patients with atrial and ventricular septal defects. The constituent ratio of maternal etiology classification in the mild, moderate, and severe PH groups was statistically significant (p < 0.001).

### Maternal outcomes

The gestational weeks at pregnancy termination in the mild, moderate, and severe PH groups were 34.8 ± 6.6, 31.7 ± 7.6, and 30.8 ± 8.0 weeks, respectively, and there were significant differences among the three groups (p < 0.05). In the mild PH group, 63.4% (52/82) of the women chose cesarean section, 23.2% (19/82) received general anesthesia, and 40.2% (33/82) received intraspinal anesthesia; in the moderate PH group, 79.4% (27/34) chose cesarean section, 38.2% (13/34) received general anesthesia, and 41.2% (14/34) received intraspinal anesthesia; and in the severe PH group, 76.3% (29/38) chose cesarean section, 52.6% (20/38) received general anesthesia, and 23.7% (9/34) received intraspinal anesthesia. Differences in the mode of delivery (p = 0.010) were statistically significant among the PH groups with different severities (p < 0.05).

Among the 154 pregnant women, one patient in the mild PH group and four patients in the severe PH group died; the maternal mortality rate was 3.2% (5/154). The deaths occurred within seven days after delivery, and there were no deaths during pregnancy. In the mild PH group, the PASP of the deceased patient was 43 mmHg, serious thrombotic events occurred before and after delivery, and the patient died due to ineffective rescue on the sixth day after abortion. Three of the four patients in the severe PH group who died were diagnosed with iPAH. The women were in extremely critical condition and were transferred from the emergency departments of other hospitals. At admission, their PASP values were 75 mmHg, 90 mmHg, and 152 mm Hg, respectively. No regular prenatal examination was performed during pregnancy. Postpartum hemorrhage and PH crisis occurred; although the women were actively resuscitated, their conditions could not be reversed, and they died. The fourth patient in the severe PH group who died was diagnosed with congenital heart disease. On admission, the patient's PASP was 148 mmHg. The patient gave birth in another hospital, and postpartum hemorrhage occurred. When she was transferred to the hospital, she was in an extremely critical state, targeted drug treatment could not be administered, and she died due to ineffective rescue.

In this study, there were 23 cases of heart failure. Patients in the mild, moderate, and severe PH groups accounted for 30.4% (7/23), 21.7% (5/23), and 47.8% (11/23) of heart failure patients, respectively. The difference among the groups was statistically significant (p < 0.05). Four patients (4/154) had a pulmonary embolism. The primary diseases were systemic lupus erythematosus (n = 2) and iPAH (n = 2), including one case in the moderate PH group (1/34) and three cases in the severe PH group (3/38). The difference among the three groups was statistically significant (p = 0.029). There were no significant differences in the incidence of complications, such as postpartum uterine asthenia, postpartum hemorrhage, and multiple organ dysfunction, among the three groups (p > 0.05).

### Fetal/neonatal outcomes

In this study, the numbers of premature infants (< 37 weeks) with mothers in the mild, moderate, and severe PH groups were 27 (32.9%), 18 (52.9%), and 21 (55.3%), respectively (Table 2). There were significant differences among the three groups (p < 0.05). Low-birth-weight (< 2,500 g) infants with mothers in the mild, moderate and severe PH groups numbered 18 (22%), 12 (35.3%), and 14 (36.8%), respectively, and no significant differences were observed among the groups (p > 0.05). Very-Low-Birth-Weight (VLBW) (1,500‒2,500 g) infants with mothers in the mild, moderate and severe PH groups numbered 5 (6.1%), 7 (20.6%), and 8 (21.1%), respectively, and significant differences were observed among the groups (p < 0.05). Extremely Low Birth Weight (ELBW) infants with mothers in the mild, moderate, and severe PH groups numbered 2 (2.4%), 1 (2.9%), and 2 (5.3%), respectively, and no significant differences were observed among the groups (p > 0.05). Small for Gestational Age (SGA) infants (fetal/neonatal weight of SGA < 1 0%) with mothers in the mild, moderate, and severe PH groups numbered 42 (51.2%), 25 (73.5%), and 27 (71.1%), respectively, and significant differences were observed among the groups (p < 0.05).

**Table 2:** Fetal/neonatal outcomes of pregnancies complicated by PH.

Fetal/neonatal outcome	Total (n = 154)	Mild PH group (n = 82)	Moderate PH group (n = 34)	Severe PH group(n = 38)	p-value*
	n (%)				
Fetal death (in utero)	7 (4.5)	4 (4.9)	0 (0)	3 (6.2)	0.588^b^
Neonatal death (≤28 days)	3 (1.9)	0 (0)	1 (2.9)	2 (5.3)	0.094^b^
Fetal/neonatal death	10 (6.5)	4 (4.9)	1 (2.9)	5 (13.2)	0.209^b^
Other complications					
Premature delivery (< 37 weeks)	66 (42.9)	27 (32.9)	18 (52.9)	21 (55.3)	0.029^a^
Moderately premature delivery (< 34 weeks)	33(21.4)	8 (9.8)	10 (29.4)	15 (39.5)	<0.001^a^
Therapeutic abortion	15 (9.7)	6 (7.3)	5 (14.7)	4 (10.5)	0.429^b^
LBW (1,500‒2,500 g)	44 (28.6)	18 (22)	12 (35.3)	14 (36.8)	0.15^a^
VLBW (1,000‒1,500 g)	20 (13.0)	5 (6.1)	7 (20.6)	8 (21.1)	0.018^b^
ELBW (< 1,000 g)	5 (3.2)	2 (2.4)	1 (2.9)	2 (5.3)	0.848^b^
SGA	94 (61.0)	42 (51.2)	25 (73.5)	27 (71.1)	0.028^a^
Neonatal unit admission	39 (25.3)	18 (22)	9 (26.5)	12 (31.6)	0.521^a^

LBW, Low Birth Weight (1,500‒2,500 g); VLBW, Very Low Birth Weight (1,000‒1,500 g); ELBW, Extremely Low Birth Weight (< 1000 g); SGA, Small for Gestational Age.

*p-value of difference among the mild, moderate, and severe PH groups.

a Chi-square test.

b Fisher's Exact Test.

There were three neonatal deaths, and the neonatal mortality rate was 1.9%. Among the neonatal deaths, there were no cases in the mild PH group, one case in the moderate PH group (2.9%), and two cases in the severe PH group (5.3%), and no significant differences were observed among the three groups (p > 0.05). There were 7 cases (4.5%, 7/154) of fetal death in utero, and three mothers were diagnosed with systemic lupus erythematosus.

### Association between PASP and maternal and fetal/neonatal death

The authors revealed the association between PASP and maternal mortality through multivariate logistic regression analysis (Table 3). The Odds Ratio (OR) (95% Confidence Interval ‒ 95% CI) of maternal death is presented for the severe PH group compared to the mild-moderate PH group. In the unadjusted model, each 10 mmHg increase in participants' PASP had a 49% increased risk of maternal death (OR = 1.49 [95% CI 1.15∼1.94]), p < 0.05. Participants who had severe PH had a 12.53-fold increased risk compared to participants with mild-moderate PH (OR = 13.53 [95% CI 1.46∼125.13]), p < 0.05. After adjustment for confounding factors, such as age, gestational weeks, systolic blood pressure, BMI, mode of delivery and anesthesia, each 10 mmHg increase in participants' PASP was associated with a 50% increased risk of maternal death (OR = 1.50 [95% CI 1.12∼2.00]), p < 0.05. Participants who had severe PH had a 20.21-fold increased risk compared to participants with mild-moderate PH (OR = 21.21 [95% CI 1.7∼264.17]), p < 0.05.

**Table 3:** Association between PASP and maternal death in the multiple logistic regression model.

			Unadjusted		Model 1		Model 2		Model 3	
Variable	Total (n)	Event n (%)	Crude OR (95% CI)	Crude (p-value)	Adj. OR (95% CI)	Adj. (p-value)	Adj. OR (95% CI)	adj. (p-value)	Adj. OR (95% CI)	adj. (p-value)
PASP per 10 mmHg	154	5 (3.2)	1.49 (1.15∼1.94)	0.003^a^	1.51 (1.14∼2)	0.004^a^	1.56 (1.16∼2.09)	0.003^a^	1.5 (1.12∼2)	0.006^a^
Mild-Moderate PH (PASP 36‒69 mmHg)	116	1 (0.9)	1 (Ref)		1 (Ref)		1 (Ref)		1 (Ref)	
Severe PH (PASP ≥70 mmHg)	38	4 (10.5)	13.53 (1.46∼125.13)	0.022^a^	16.5 (1.37∼198.41)	0.027^a^	23.74 (1.99∼283.91)	0.012^a^	21.21 (1.7∼264.17)	0.018^a^

OR, Odds Ratio; 95% CI, 95% Confidence Interval; Model 1, Adjusted for age + gestational weeks + systolic blood pressure + Body Mass Index (BMI); Model 2, Adjusted for Model 1 + Vaginal delivery + Cesarean section; Model 3, Adjusted for Model 2 + General anesthesia + Spinal anesthesia.

a Multivariate logistic regression analysis.

As shown in Table 2, there was no significant difference in fetal/neonatal mortality among the mild, moderate, and severe PH groups (p > 0.05).

### Twelve-month postpartum follow-ups

A total of 131 patients (85.1%) were followed up at 12 months postpartum, and 32 patients (20.8%) had PASP values that remained abnormal. Nine patients (5.8%) underwent cardiac surgery after delivery; 3 (1.9%) women died within 12 months after delivery, and all of them were included in the severe PH group; and one (0.6%) infant died within 12 months, and the infant's mother was included in the moderate PH group.

## Discussion

PH is a syndrome of pulmonary vascular resistance and elevated PASP caused by various etiologies and pathogeneses. Pregnancy complicated by PH is related to many factors and has a high mortality rate. In this study, the maternal mortality rate was 3.2% at 7 days postpartum. The maternal mortality rate of pregnancy with PH in this study is similar to that reported by Karen Sliwa et al.^6^ However, the 7-day postpartum maternal mortality rate in the severe PH group was 10.5%. The risk of maternal death in the severe PH group was significantly higher than that in the mild-moderate group. This may be because the severity of elevated PASP may be related to more serious primary diseases.

With the use of broad-spectrum antibiotics, the incidence rate of rheumatic heart disease decreases annually. However, according to the clinical classification of 154 pregnant patients with PH in this study, the most common clinical type was LHD-PH (40.3%, 62/154), which is consistent with the epidemiological data reported in the Chinese guidelines for the diagnosis and treatment of pulmonary hypertension and the ESC/ERS guidelines for the diagnosis and treatment of pulmonary hypertension.^9^

In the early stages of pregnancy, patients with mild PH may not have obvious symptoms, while patients with moderate or severe PH may have clinical symptoms, such as chest tightness, palpitation, and dyspnea. However, due to the continuous increase in pulmonary vascular remodeling and pulmonary vascular resistance in women with PH, it is difficult for their bodies to adapt to changes in blood volume and cardiac output through self-regulation. This results in a further increase in average pulmonary artery pressure, an increase in right heart afterload, compensatory hypertrophy of the right ventricle, and gradual deterioration of cardiac function, causing right heart failure.^15^^,^^16^ New York Heart Association (NYHA) grading of cardiac function is an important indicator of disease progression, and the survival time of patients with cardiac function grades I–II is longer than that of patients with cardiac function grades III or IV.^17^ This study showed that the incidence of heart failure in the severe PH group was significantly higher than that in the other two groups. Late pregnancy, delivery, and 3-days postpartum are the most dangerous periods for pregnant women with PH.^7^ The five maternal deaths in this study occurred within 7-days postpartum. This is because the higher the pulmonary artery pressure is, the worse the pulmonary vascular compensation ability. Postpartum uterine contraction leads to a sudden increase in venous return to the heart and cardiac output, which increases the load on the right heart. In women with severe PH, the pulmonary vessels and heart are more likely to exceed their compensatory capacity, leading to the development of heart failure or PH crisis. This suggests that monitoring should be strengthened and close attention should be given to changes in the conditions of pregnant patients with PH after delivery; intervention measures should be performed as soon as possible to avoid death after pregnancy and childbirth.^18^

The delivery time of pregnant women with PH can be determined according to the severity of the disease, the PASP value, and gestational age. If cardiac function deteriorates during pregnancy, it is best to terminate the pregnancy before 32 weeks. Currently, there is no consensus on the best mode of delivery for pregnant women with PH.^7^ In this study, the proportion of cesarean sections in the mild, moderate, and severe PH groups was significantly higher than that of vaginal delivery. The cesarean section rate was 70.1%; therefore, cesarean section was the preferred delivery method in this study. The cesarean section offers faster delivery, reduces the risk of postpartum hemorrhage and hemodynamic disorders caused by prolonged uterine contractions, and reduces uterine contraction pain in pregnant women. Intraspinal anesthesia is a safe method, but experienced medical institutions also recommend general anesthesia for cesarean section in pregnant women with severe PH.^9^ Whether cesarean section under general anesthesia is better than intraspinal anesthesia is still controversial and needs further research.

Different severities of PH could affect perinatal complications and outcomes. The incidence of fetal or neonatal death is approximately 10%.^2^, ^3^, ^4^, ^5^, ^6^ In this study, the incidence of premature birth was higher in the severe PH group than in the other two groups. It is well known that adverse fetal outcomes are related to maternal hypoxemia. Chronic maternal hypoxia can easily lead to intrauterine fetal death.^19^ The relationship among pregnancies complicated by PH, placental perfusion, and adverse fetal/neonatal outcomes is worthy of further study.

This study has several limitations. First, the present research subjects were from a severe maternal care center in South China. Therefore, this was a single-center study with a limited number of subjects, and studies with more centers and a larger sample size are required for further verification of the results. Second, few patients were diagnosed with PH by RHC before pregnancy, and most patients were diagnosed with PH by echocardiography during pregnancy. Strictly speaking, RHC is the gold standard for diagnosing PH, but pregnant women exposed to electromagnetic radiation are prone to risks such as fetal malformation and miscarriages. Therefore, it is not recommended that RHC be used as a perinatal monitoring method for pregnant women.

In this study, the authors found that the risk of maternal mortality in the severe PH group was significantly higher than that in the mild-moderate group, highlighting the importance of pulmonary artery pressure screening before pregnancy, early advice on contraception, and multidisciplinary care.

## Research ethics and patient consent

The study was conducted according to the guidelines of the Declaration of Helsinki and approved by the institutional review board of the Third Affiliated Hospital of Guangzhou Medical University, with ethics approval ID [2022] NO. 030. (Protocol code 30 and date of approval 24 April 2022). Written informed consent was obtained from patients or their representatives for participation in this study. All methods were performed in accordance with relevant guidelines and regulations.

## Data availability statement

The raw data supporting this study can be requested via the corresponding author.

## Authors' contributions

CTL and LCW are joint first authors. YMG designed the study. GYL, YWH, JYC, SYJ, and DJC collected the data. GYL and YWH were involved in data cleaning, follow-up, and verification. JYC, SYJ, and DJC analyzed the data. CTL drafted the manuscript. YMG contributed to the interpretation of the results and critical revision of the manuscript for important intellectual content and approved the final version of the manuscript. All authors have read and approved the final manuscript. YMG is the study guarantor.

## Conflicts of interest

The authors declare no conflicts of interest.
